# Pepper CaREL1, a ubiquitin E3 ligase, regulates drought tolerance via the ABA-signalling pathway

**DOI:** 10.1038/s41598-017-00490-4

**Published:** 2017-03-28

**Authors:** Chae Woo Lim, Chanmi Park, Jung-Hyun Kim, Hyunhee Joo, Eunji Hong, Sung Chul Lee

**Affiliations:** 1 Department of Life Science (BK21 program), Chung-Ang University, Seoul, 06974 Republic of Korea; 2 Department of Physical Education, Chung-Ang University, Seoul, 06974 Republic of Korea

## Abstract

Drought stress conditions in soil or air hinder plant growth and development. Here, we report that the hot pepper (***C*** *apsicum* ***a*** *nnuum*) RING type E3 Ligase 1 gene (*CaREL1*) is essential to the drought stress response. *CaREL1* encodes a cytoplasmic- and nuclear-localized protein with E3 ligase activity. *CaREL1* expression was induced by abscisic acid (ABA) and drought. *CaREL1* contains a C3H2C3-type RING finger motif, which functions in ubiquitination of the target protein. We used *CaREL1*-silenced pepper plants and *CaREL1*-overexpressing (OX) transgenic Arabidopsis plants to evaluate the *in vivo* function of CaREL1 in response to drought stress and ABA treatment. *CaREL1*-silenced pepper plants displayed a drought-tolerant phenotype characterized by ABA hypersensitivity. In contrast, *CaREL1*-OX plants exhibited ABA hyposensitivity during the germination, seedling, and adult stages. In addition, plant growth was severely impaired under drought stress conditions, via a high level of transpirational water loss and decreased stomatal closure. Quantitative RT-PCR analyses revealed that ABA-related drought stress responsive genes were more weakly expressed in *CaREL1*-OX plants than in wild-type plants, indicating that CaREL1 functions in the drought stress response via the ABA-signalling pathway. Taken together, our results indicate that CaREL1 functions as a negative regulator of ABA-mediated drought stress tolerance.

## Introduction

Plants are sessile organisms, and hence they frequently encounter various stress conditions such as high light, extreme temperature, and water stress. Among these stress conditions, water stress in soil dramatically affects crop yield and agricultural quality worldwide. Plants alleviate the adverse effects of water stress via alteration of ion transport and regulation of stomatal aperture to reduce transpirational water loss. Abscisic acid (ABA) plays a key role in plant defence responses, especially the drought stress response. When exposed to drought stress, plants increase ABA biosynthesis and accumulation in the leaves and initiate signal transduction leading to plant defence responses^1–3^. A large number of stress-related genes involved in these defence mechanisms are regulated by ABA^4^. The ABA- and drought-signalling pathways have been extensively investigated; however, the specific mechanisms underlying the functional modifications remain unclear.

Ubiquitination with the 26S proteasome system is a common protein modification mechanism in eukaryotic cells^5, 6^. In plants, ubiquitination is necessary for various cellular signalling processes with a diverse range of targets such as transcription factors, hormone receptors, and damaged proteins^7–10^. The process of ubiquitin attachment to the target protein is conserved and involves three proteins—E1 (ubiquitin-activating enzyme), E2 (ubiquitin-conjugating enzyme), and E3 (ubiquitin ligase)^11^. Ubiquitin is composed of 76 amino acids and contains seven lysines that serve as the site of modification^12^. Ubiquitination via these enzymes conjugates single or multiple ubiquitins to the target proteins, which are recognized by the 26S proteasome for degradation. In this process, E3 ubiquitin ligase plays a major role as the target recognition component responsible for recruiting the target protein and transferring ubiquitin to the substrate. E3 ubiquitin ligase has a large number of multiple isoforms and determines the specificity of the target protein^8, 10^. E3 ubiquitin ligases are divided into two distinct superfamilies according to whether the structural domain contains a single or multi-subunit. One superfamily comprises the Really Interesting New Gene (RING), Homology to E6-AP Carboxyl Terminus (HECT), and U-box E3 ligases^13–16^. The other superfamily is composed of CULLIN4-Damaged-specific DNA binding protein1 (CUL4-DDB1), Anaphase Promoting Complex (APC), and Skp1-Cullin-F-box (SCF) E3 ligases^17–19^. The Arabidopsis genome contains more than 1,400 E3 ubiquitin ligases^10^. These include approximately 477 RING finger domain-containing E3 U-box ligases, which are subdivided into eight subgroups according to the type of RING domain^20^. To date, however, the precise function of only a few RING type E3 ligases has been elucidated.

In the present study, we identified the *C* *apsicum* *a* *nnuum* RING type E3 Ligase 1 gene (*CaREL1*), which encodes a RING type E3 ligase. We used *CaREL1*-silenced pepper plants and *CaREL1-*overexpressing (OX) transgenic Arabidopsis plants to examine the potential roles of *CaREL1* in the abiotic stress response. *CaREL1*-silenced pepper plants exhibited an ABA-hypersensitive phenotype with significantly enhanced tolerance to drought stress. On the other hand, *CaREL1*-OX Arabidopsis plants exhibited an ABA-insensitive and drought-sensitive phenotype characterized by a high transpiration rate and altered expression levels of stress-related genes. Our findings indicate that the CaREL1 RING type E3 ligase negatively regulates drought stress tolerance via the ABA-signalling pathway.

## Results

### Isolation and sequence analysis of the *CaREL1* gene

**Figure 1:**
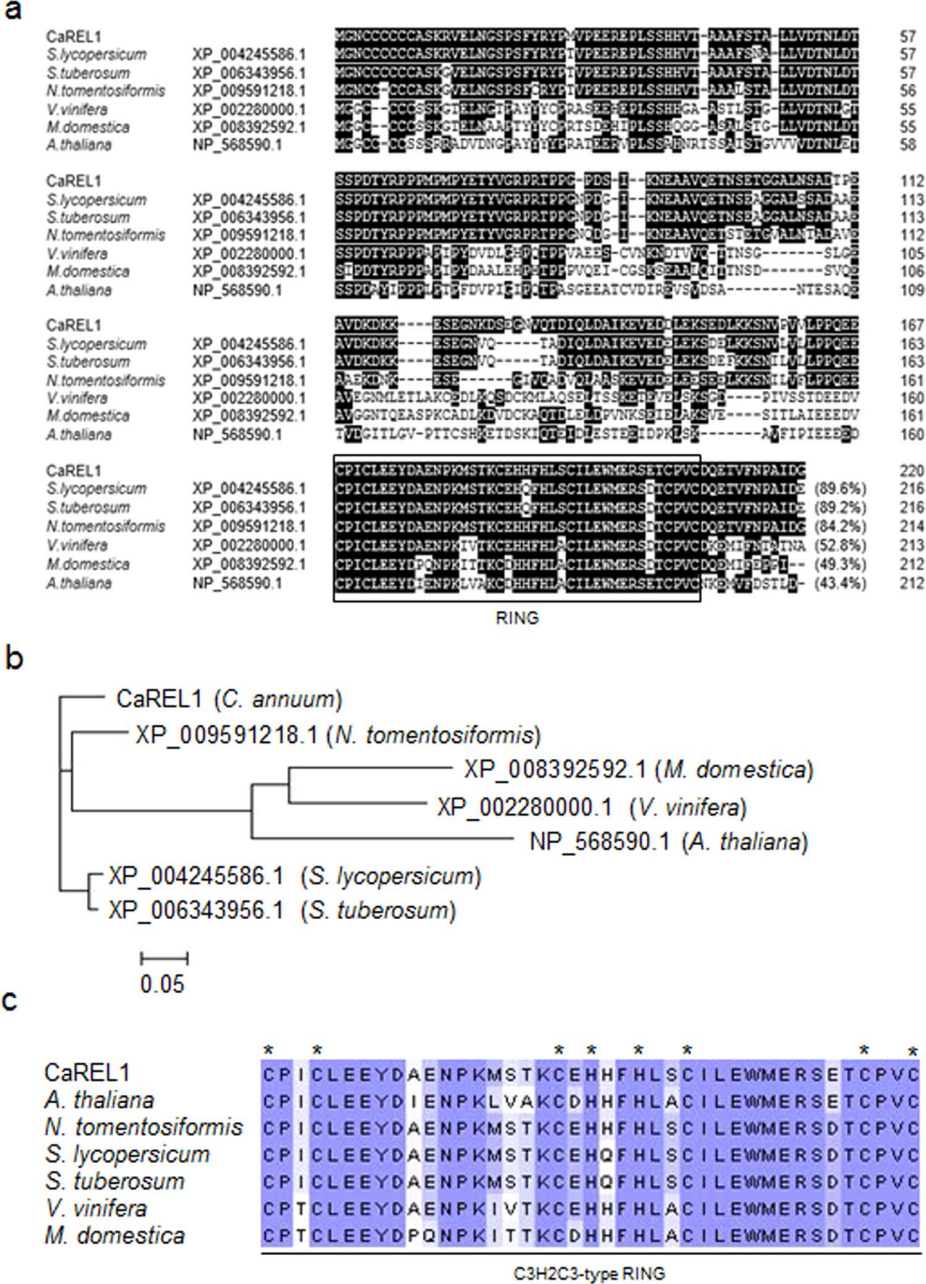
Amino acid sequence analysis of the pepper CaREL1 (*C* *apsicum* *a* *nnuum* RING type E3 Ligase 1) protein. (**a**) Comparisons of the deduced amino acid sequence of the CaREL1 protein (accession no. KU557246) with those of the *Solanum lycopersicum* (accession no. XP_004245586.1), *Solanum tuberosum* (accession no. XP_006343956.1), *Nicotiana tomentosiformis* (accession no. XP_009591218.1), *Vitis vinifera* (accession no. XP_002280000.1), *Malus domestica* (accession no. XP_008392592.1), and *Arabidopsis thaliana* (accession no. NP_568590.1) proteins. Identical amino acid residues are highlighted in black, and the box reveals the Really Interesting New Gene (RING) zinc finger domain. (**b**) Phylogenetic tree analysis of the CaREL1 protein. Blast search was performed using the deduced amino acid sequences of the *CaREL1* gene, and the top-ranked sequences from each plant species were collected. (**c**) Alignment of the RING zinc finger C3H2C3-type domain. Asterisks indicate conserved cysteine (C) and histidine (H) residues.

The *CaREL1* gene was isolated from a cDNA library constructed from ABA-treated pepper leaves by using differential hybridization^21^. The putative *CaREL1* consists of a 663-bp open reading frame encoding 220 amino acid residues with an isoelectric point (pI) of 4.52 and a calculated molecular mass of 24.3 kDa (Fig. 1a). Multiple sequence alignment analysis revealed high amino acid sequence identity between CaREL1 (accession no. KU557246) and RING finger proteins of *Solanum lycopersicum* (accession no. XP_004245586.1, 89.6% identity), *Solanum tuberosum* (accession no. XP_006343956.1, 89.2% identity), *Nicotiana tomentosiformis* (accession no. XP_009591218.1, 84.2% identity), *Vitis vinifera* (accession no. XP_002280000.1, 52.8% identity), *Malus domestica* (accession no. XP_008392592.1, 49.3% identity), and *Arabidopsis thaliana* (accession no. NP_568590.1, 43.4% identity) (Fig. 1a and b). All of these proteins have a highly conserved C3H2C3 type RING finger domain (Fig. 1c). The RING domain of CaREL1 contains eight conserved cysteine and histidine residues, which are necessary for E3 ubiquitin ligase activity^9, 22^ (Fig. 1c).

### Subcellular localization and *in vitro* ubiquitination of CaREL1

**Figure 2:**
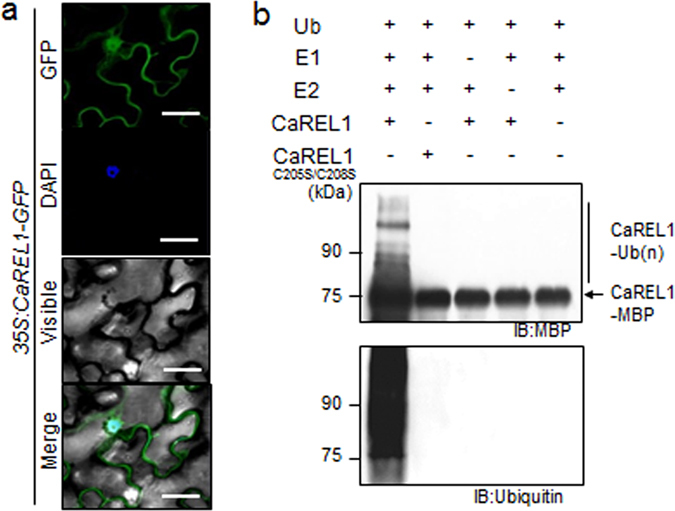
CaREL1 is localized at nucleus and has E3 ligase activity. (**a**) Subcellular localization of the 35S:*CaREL1*-GFP fusion protein in *Nicotiana benthamiana* epidermal cells. The 35S:*CaREL1*-*GFP* construct was expressed via agroinfiltration of *N*. *benthamiana* leaves and observed using a confocal laser-scanning microscope. DAPI staining was used as a marker for the nucleus. The scale bar represents 20 μm. (**b**) Auto-ubiquitination of CaREL1. In the presence of ubiquitin, E1 (UbE1), and E2 (UBHPC H5b), maltose-binding protein (MBP)–CaREL1 fusion proteins displayed E3 ubiquitin ligase activity. Detection of MBP–CaREL1 auto-ubiquitination. MBP–CaREL1^C205S/C208S^ was loaded as negative control. MBP–CaREL1 fusion proteins were detected using MBP and ubiquitin antibodies; shifted bands indicate the attachment of ubiquitin molecules.

To analyse the subcellular localization of the CaREL1 protein in plant cells, we fused the reporter gene of the green fluorescent protein (GFP) to the *CaREL1* coding region under the control of 35S promoter, to produce 35S:*CaREL1-GFP* fusion. The 35S:*CaREL1-GFP* fusion protein generated GFP signals in the cytoplasm and nucleus of *Nicotiana benthamiana* epidermal cells (Fig. 2a). We used DAPI staining as a control and observed blue signals in the nucleus. Our results suggest that the CaREL1 protein functions in the cytoplasm and nucleus.

The CaREL1 protein contains a C3H2C3-type RING domain, which has E3 ligase activity^7, 23, 24^. We investigated whether CaREL1 has E3 ligase activity by conducting an *in vitro* self-ubiquitination assay with maltose-binding protein tagged CaREL1 (MBP::CaREL1). The purified MBP::CaREL1 fusion protein was incubated with or without ubiquitin, E1, and E2 and separated using SDS-PAGE gel. The ubiquitinated protein was detected using western blot analysis with anti-MBP and anti-ubiquitin antibodies (Fig. 2b). In the presence of ubiquitin, E1, and E2 enzymes, we detected additional higher molecular-weight bands. However, a catalytic dead mutant of CaREL1, CaREL1^C205S/C208S^, did not show self-ubiquitination activity. These data indicate that CaREL1 has E3 ligase activity.

### Induction of the *CaREL1* gene in pepper tissues

We performed RT-PCR analysis to determine whether the *CaREL1* gene is constitutively expressed in different tissues of pepper plants (Supplementary Fig. S1). *CaREL1* was expressed in root, stem, leaf, and flower tissues; moreover, high transcript levels were found in root and flower tissues. *CaREL1* was isolated from drought-treated leaves; hence, we examined whether the expression of this gene is induced by abiotic stresses such as ABA and drought (Fig. 2c). In ABA-treated leaf tissues, *CaREL1* expression was not upregulated at 0–12 h; however, high transcript levels were detected at 24 h. Moreover, the steady-state levels of *CaREL1* transcripts were slightly upregulated by drought treatment. Our results indicate that *CaREL1* functions in the ABA-signalling and drought stress responses.

### ABA hypersensitivity and enhanced drought tolerance of *CaREL1*-silenced pepper plants

**Figure 3:**
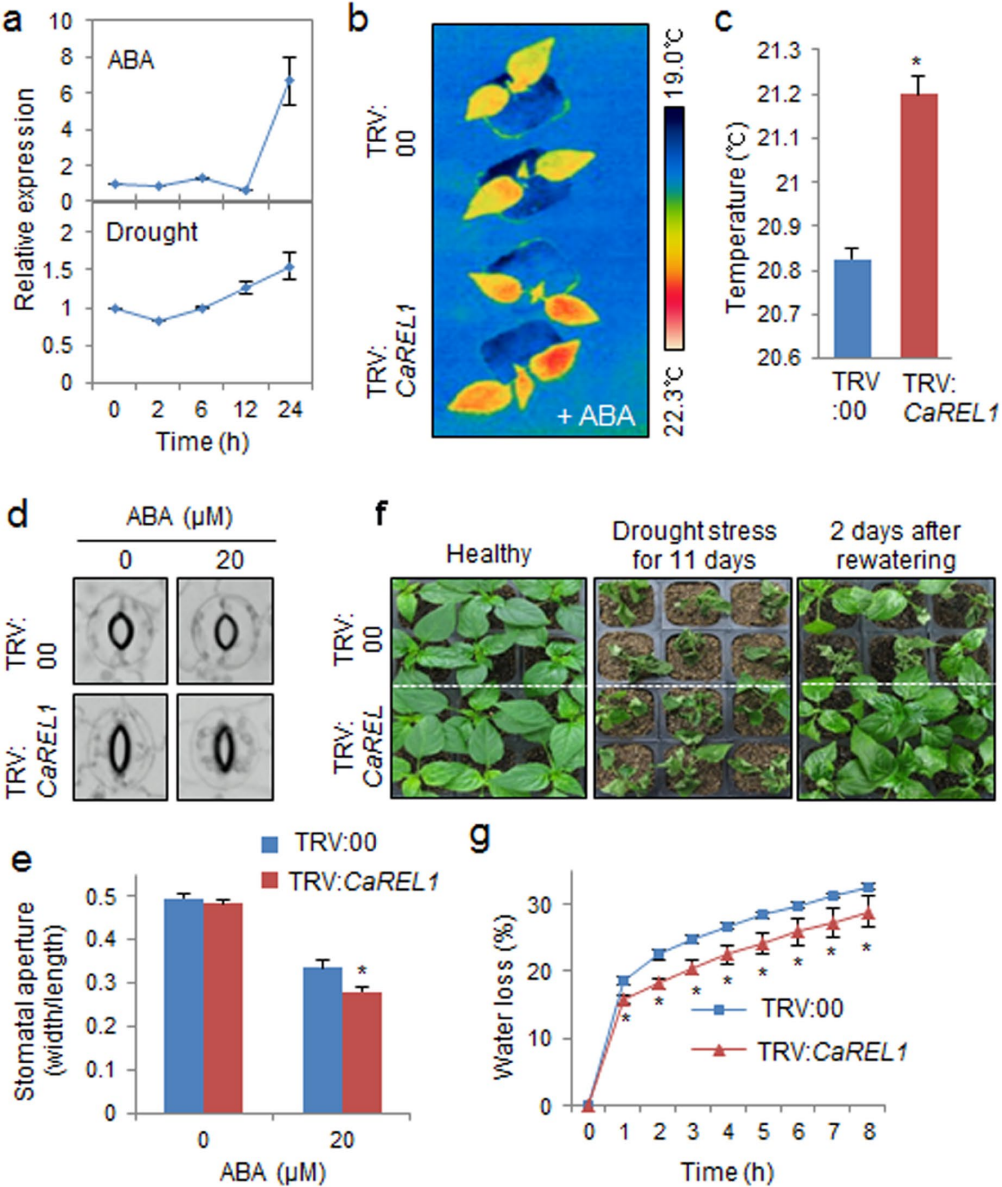
Enhanced tolerance of *CaREL1-*silenced pepper plants to drought stress. (**a**) qRT-PCR analysis of *CaREL1* gene expression in pepper leaves 0–24 h after treatment with 50 μM abscisic acid (ABA) or drought. (**b** and **c**) Increased leaf temperatures of *CaREL1-*silenced pepper plants in response to ABA treatment. Leaf temperatures after treatment with 50 μM ABA were measured using thermal imaging and representative images were taken (**b**). Data represent the mean ± standard error of three independent experiments (**c**). (**d** and **e**) Stomatal apertures in control and *CaREL1*-silenced pepper plants treated with ABA. Leaf peels were harvested from 4-week-old plants of each line and incubated in SOS buffer containing 0 μM or 20 μM ABA. Stomatal apertures were measured under the microscope and representative images were taken (**d**). Data represent the mean ± standard error of three independent experiments (**e**). (**f**) The drought-tolerant phenotype of *CaREL1*-silenced pepper plants. Control and *CaREL1*-silenced pepper plants were grown in pots for 6 weeks under well-watered conditions. Thereafter, watering was withheld for 11 days, followed by re-watering for 2 days. (**g**) Survival rates of control and *CaREL1*-silenced pepper plants after 2 days of re-watering. Data represent the mean ± standard error of three independent experiments, each evaluating 20 plants. (**h**) Transpirational water loss from the leaves of empty vector control and *CaREL1*-silenced pepper plants at various times after detachment of leaves.

To investigate the response of *CaREL1* to abiotic stress, we used a virus-induced gene silencing (VIGS) system with the tobacco rattle virus (TRV) vector to analyse loss-of-function CaREL1 mutants^25^. *CaREL1* transcript accumulation was approximately 50% lower in *CaREL1*-silenced pepper plants than in control plants (Supplementary Fig. S2). Abiotic stress and ABA signals share common signal transduction components; however, drought stress signalling has ABA-dependent and ABA-independent pathways^26^. *CaREL1* transcripts were accumulated after ABA and drought stress treatments, suggesting that *CaREL1* functions in stress-induced signalling. Thus, we compared the ABA sensitivity of *CaREL1-*silenced pepper plants with that of control plants (Fig. 3a–e). First, we measured the leaf temperature—as an indication of stomatal aperture—in fully expanded leaves of control and *CaREL1-*silenced pepper plants after exposure to 50 μM ABA (Fig. 3a and c). *CaREL1*-silenced pepper plants had higher leaf temperatures than control plants. Stomatal opening leads to an increase in evaporative cooling and therefore to decrease in leaf temperatures^27, 28^; thus, we predicted that ABA-treated *CaREL1*-silenced pepper plants would show increased stomatal closure relative to control plants. We exposed control and *CaREL1-*silenced pepper plants to 20 μM ABA for 3 h and measured the stomatal pore sizes. Consistent with the leaf temperatures, the stomatal apertures were smaller in *CaREL1-*silenced pepper plants than in control plants (Fig. 3d and e).

Under well-watered conditions, we observed no phenotypic differences between *CaREL1-*silenced pepper plants and control plants (Fig. 3f, left panel). However, when plants were subjected to drought stress by withholding watering for 11 days, followed by re-watering for 2 days, the *CaREL1*-silenced pepper plants exhibited a drought-tolerant phenotype (Fig. 3f, middle and right panels). Moreover, after re-watering, 87.5% of the *CaREL1-*silenced pepper plants resumed their growth, whereas the survival rate of control plants was only 37.5% (Fig. 3f). Hence, *CaREL1* expression led to enhanced drought tolerance. To ascertain whether the enhanced drought tolerance of *CaREL1*-silenced pepper plants was caused by increased water retention in leaf tissues, we monitored the transpirational water loss by measuring the fresh weight of leaves 8 h after detachment (Fig. 3g). Consistent with the drought-tolerant phenotype, the leaf water content was higher in *CaREL1*-silenced pepper than in control plants. Our results indicate that the enhanced drought tolerance of *CaREL1*-silenced pepper was derived from an increase in water retention capacity owing to ABA hypersensitivity.

### ABA hyposensitivity of *CaREL1*-OX plants

**Figure 4:**
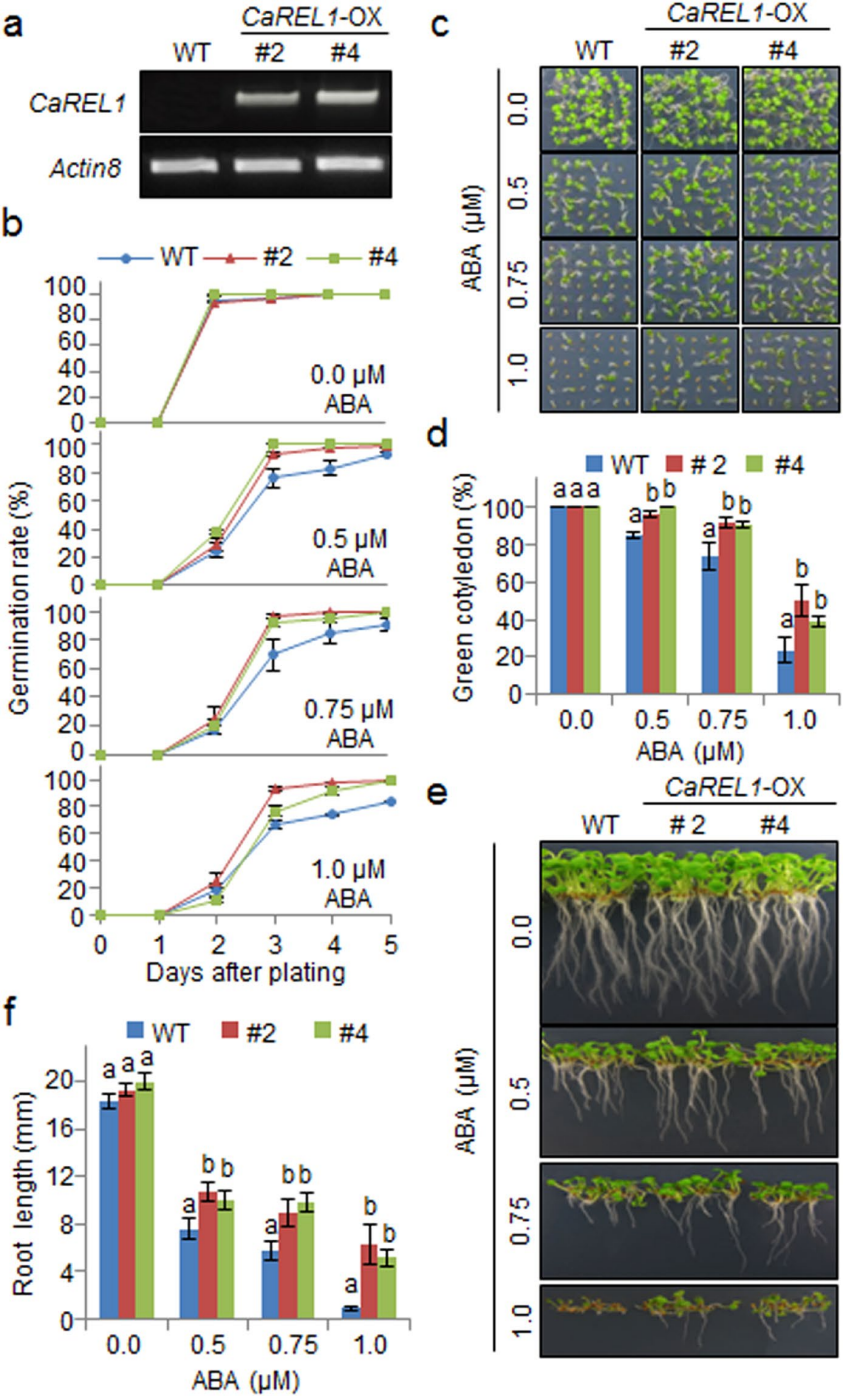
Reduced sensitivity of *CaREL1-*OX transgenic Arabidopsis lines to ABA. (**a**) Reverse transcription-polymerase chain reaction (RT-PCR) analysis of *CaREL1* expression in wild-type and *CaREL1*-OX plants. *Actin8* was used as an internal control gene. (**b**) Seed germination of wild-type and *CaREL1-*OX plants in response to ABA for 0–5 days after treatment. Seeds were germinated on 0.5× MS agar plates containing 0.0 μM, 0.5 μM, 0.75 μM, or 1.0 μM ABA. (**c**) Growth of wild-type and *CaREL1-*OX seedlings on 0.5× MS agar plates containing 0.0 μM, 0.5 μM, 0.75 μM, or 1.0 μM ABA. Representative photographs were taken 10 days after plating. (**d**) Quantification of green cotyledons in the wild-type and each mutant line was performed 10 days after plating on 0.5× MS agar plates containing 0.0 μM, 0.5 μM, 0.75 μM, or 1.0 μM ABA. Data represent the mean ± standard error of three independent experiments, each evaluating 30 seeds. (**e**) Root elongation of wild-type and transgenic plants in response to ABA. (**f**) The root length of each plant was measured 10 days after plating. Data represent the mean ± standard error of three independent experiments. Different letters indicate significant differences between wild-type and transgenic lines (*P* < 0.05; ANOVA followed by Fisher’s LSD test).

**Figure 5:**
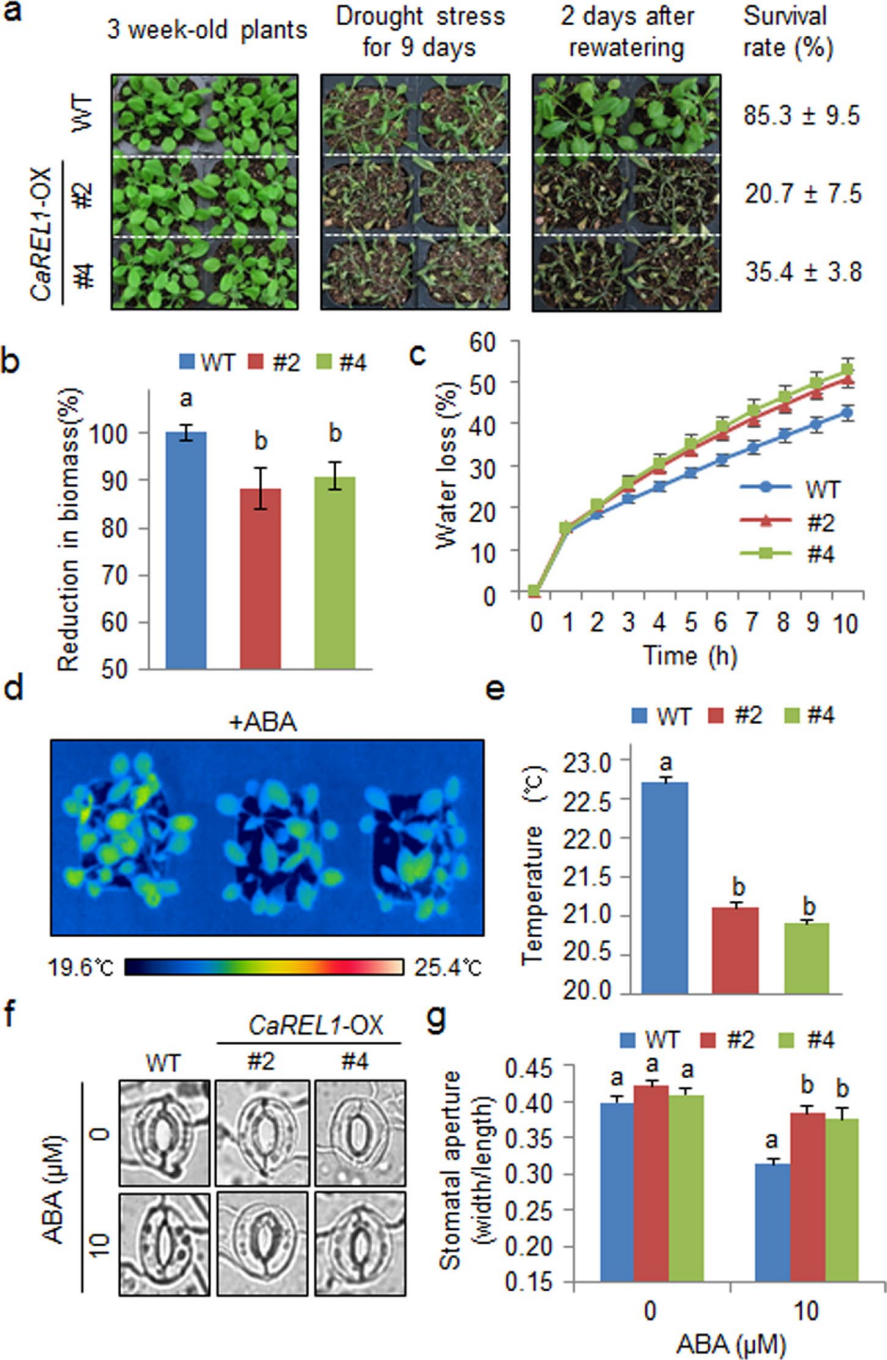
Reduced tolerance of *CaREL1-*OX plants to drought stress. (**a**) Drought tolerance of *CaREL1-*OX transgenic plants. Three-week-old wild-type and transgenic plants were subjected to drought stress by withholding water for 9 days and then re-watering for 2 days. Survival rates of plants were measured after re-watering. (**b**) Reduced biomass of *CaREL1-*OX plants. The plant biomass was measured after re-watering. (**c**) Transpirational water loss from the leaves of wild-type and transgenic plants at various times after detachment of leaves. (**d** and **e**) Decreased leaf temperatures of *CaREL1-*OX plants in response to ABA treatment. Leaf temperatures of plants treated with 50 μM ABA were measured using thermal imaging and representative images were taken (**d**). Data represent the mean ± standard error of three independent experiments (**e**). (**f** and **g**) Stomatal apertures in wild-type and *CaREL1*-OX plants treated with ABA. Leaf peels were harvested from 3-week-old plants of each line and incubated in SOS buffer containing 0 μM or 20 μM ABA. Stomatal apertures were measured under the microscope and representative images were taken (**e**). Data represent the mean ± standard error of three independent experiments (**f**), each evaluating 20 plants. Different letters indicate significant differences between wild-type and transgenic lines (*P* < 0.05; ANOVA followed by Fisher’s LSD test).

To further examine the *in vivo* function of *CaREL1*, we generated Arabidopsis transgenic plants constitutively expressing *CaREL1* (CaMV *35S*:*CaREL1*). We performed RT-PCR analysis to examine the expression levels of two independent T_3_ homozygous transgenic progeny (*CaREL1-*OX) (Fig. 4a). Under normal growth conditions, we observed no phenotypic differences between *CaREL1-*OX plants and wild-type plants, suggesting that conferred expression of *CaREL1* does not influence normal growth and development (Figs 4 and 5). To investigate whether conferred expression of *CaREL1* affects the ABA response, we analysed the ABA sensitivity of wild-type and *CaREL1-*OX plants during the germination and seedling stages (Fig. 4). We germinated seeds on growth media supplemented with various ABA concentrations (0.0 μM, 0.5 μM, 0.75 μM, or 1.0 μM). In the absence of ABA, we determined no significant differences in germination rates between wild-type and *CaREL1*-OX seeds (Fig. 4b). In the presence of 0.5 μM ABA, wild-type and *CaREL1*-OX seeds showed germination rates of almost 100% after 5 days; however, *CaREL1*-OX seeds germinated faster than wild-type seeds. These differences were more distinguishable in the presence of higher ABA concentrations (Fig. 4b). To analyse the ABA response at the seedling stage, we measured seedling establishment and root growth in the presence of various ABA concentrations (Fig. 4c–f). After 5 days, seedling establishment was higher in *CaREL1*-OX plants than in wild-type plants (Fig. 4c and d). In addition, primary root growth of *CaREL1*-OX plants was less impaired than that of wild-type plants (Fig. 4e and f). In the absence of ABA, we determined no significant differences in the primary root lengths between wild-type and *CaREL1*-OX plants. However, in the presence of various ABA concentrations, the primary roots of *CaREL1-*OX plants were significantly longer than those if wild-type plants. Our data indicate that altered expression of *CaREL1* affects ABA sensitivity in Arabidopsis.

### Reduced drought tolerance of *CaREL1*-OX plants

*CaREL1*-silenced pepper plants and *CaREL1*-OX Arabidopsis plants showed drought-tolerant and ABA-hyposensitive phenotypes, respectively (Figs 3 and 4); hence, we predicted that *CaREL1*-OX plants would show an altered drought stress response. We compared the phenotypes of *CaREL1*-OX plants in response to drought and ABA stresses. Under favourable growth conditions, we observed no phenotypic differences between *CaREL1*-OX and wild-type plants (Fig. 5a, left panel). However, when watering was withheld for 9 days, followed by re-watering for 2 days, *CaREL1*-OX plants displayed a more wilted phenotype than wild-type plants (Fig. 5a, middle and right panels). We measured the survival rates of *CaREL1*-OX and wild-type plants after re-watering and found that 85% of wild-type plants survived, whereas the survival rates of *CaREL1*-OX #2 and *CaREL1*-OX #4 plants were only 20% and 35%, respectively (Fig. 5a). Moreover, the reduction in biomass was higher in transgenic plants than in wild-type plants (Fig. 5b). Hence, conferred expression of *CaREL1* contributes to the impaired drought tolerance of *CaREL1-*OX plants. To examine whether the altered phenotype exhibited by *CaREL1-*OX plants in response to drought stress is derived from reduced capacity for water retention, we monitored the transpirational water loss by measuring the fresh weight of detached leaves (Fig. 5c). Consistent with the drought-sensitive phenotype, the leaf water content was lower in the leaf tissues of *CaREL1-*OX plants than in those of wild-type plants.

Several studies have suggested that increased stomatal closure in response to ABA leads to a reduction in water loss via decreased transpiration. Therefore, we examined whether conferred expression of *CaREL1* influences ABA-mediated stomatal closure. First, we exposed wild-type and transgenic plants to 50 μM ABA and measured the leaf temperatures. After 3 h, the leaf temperatures of *CaREL1-*OX plants were lower than those of wild-type plants (Fig. 5d and e). Next, we measured the stomatal apertures in the presence and absence of ABA treatment (Fig. 5f and g). In the absence of ABA treatment, the stomatal apertures did not differ significantly between wild-type and *CaREL1-*OX plants. However, after 4 h of exposure to 20 μM ABA, the stomatal apertures were larger in the leaves of transgenic plants than in the leaves of wild-type plants. Our results indicate that hyposensitivity to ABA in the guard cells of *CaREL1-*OX plants may lead to decreased water retention and therefore reduced drought tolerance.

**Figure 6:**
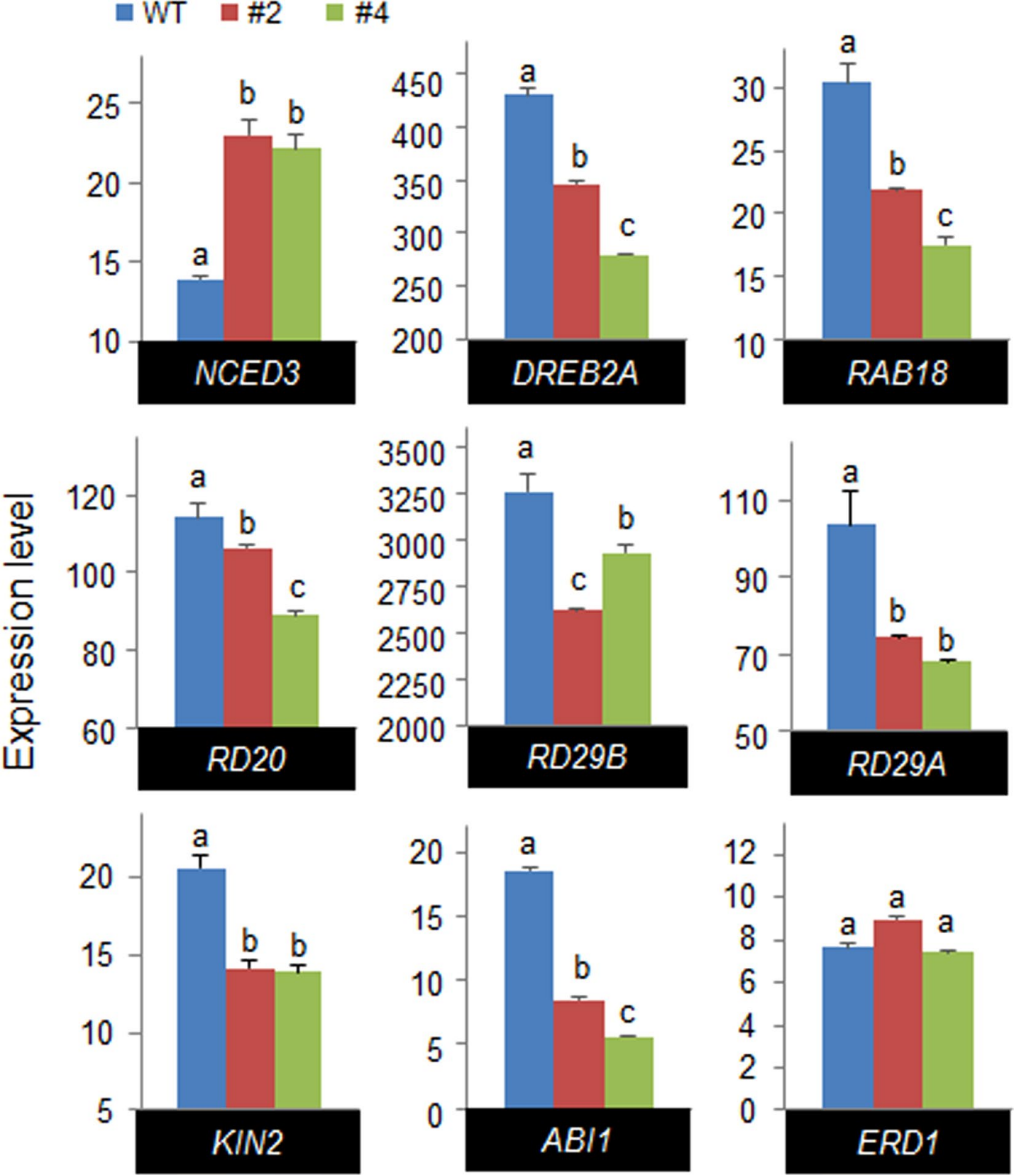
Expression of drought stress-responsive genes in *CaREL1*-OX plants. *CaREL1*-OX plants were exposed to drought stress and the expression levels of ABA-dependent stress-related genes were measured 3 h after detachment using quantitative reverse transcription-polymerase chain reaction (qRT-PCR) analysis. The relative expression levels (∆∆CT) of each gene were normalized to the geometric mean of *Actin8* as an internal control gene. Data represent the mean ± standard error of three independent experiments. Different letters indicate significant differences between wild-type and transgenic lines (*P* *<* 0.05; ANOVA followed by Fisher’s LSD test).

The expression of stress-responsive genes is associated with stress tolerance. Hence, we examined whether several drought-inducible genes are directly or indirectly regulated by CaREL1. We subjected wild-type and *CaREL1*-OX plants to dehydration stress and performed qRT-PCR analysis of leaves 3 h after detachment (Fig. 6). Induction of *NCED3*, which is associated with ABA synthesis, was significantly higher in the leaves of *CaREL1-*OX plants than in the leaves of wild-type plants. In addition, we observed lower accumulation of various stress-responsive genes, including *DREBA*, *RAB18*, *RD20*, *RD29B*, *RD29A*, and *KIN2*, in the leaves of *CaREL1-*OX plants than in the leaves of wild-type plants. Our data indicate that the expression level of *CaREL1* not only influences ABA biosynthesis, but is also involved in modulating the response to drought stress. CaREL1 may function as a negative regulator of the drought stress response via ABA-dependent signalling. Thus, we examined whether the drought stress-responsive function of CaREL1 is ABA-dependent or ABA-independent, by measuring the expression levels of *ABI1* and *ERD1* in the leaves of wild-type and *CaREL1*-OX plants. Previous studies showed that ABI1 and ERD1 function in the drought stress response via ABA-dependent and ABA-independent pathways, respectively^2, 29^. Three hours after detachment, the levels of *ABI1* transcripts were significantly lower in the leaves of transgenic plants than in the leaves of wild-type plants; however, the levels of *ERD1* transcripts did not differ significantly between the leaves of transgenic and wild-type plants (Fig. 6). Our data indicate that *CaREL1* expression negatively influences the expression levels of drought-inducible marker genes via the ABA-dependent pathway, and this contributes to the reduced drought tolerance of *CaREL1-*OX plants.

**Figure 7:**
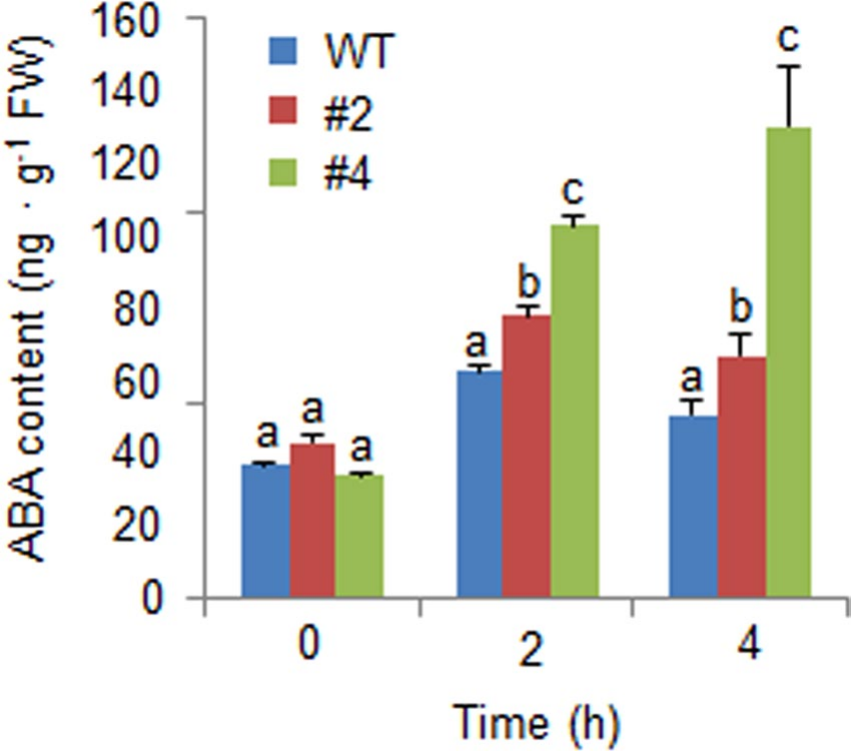
Increased accumulation of ABA in *CaREL1-*OX plants exposed to drought stress. The ABA contents in the leaf tissues were measured 0 h, 2 h, and 4 h after drought stress treatment.

The expression level of *NCED3*, which is associated with ABA biosynthesis, was significantly higher in the leaves of *CaREL1-*OX plants than in the leaves of wild-type plants. To ascertain whether overexpression of *CaREL1* is associated with ABA biosynthesis, we measured the ABA contents in the leaves of wild-type and *CaREL1-*OX plants after exposure to drought stress (Fig. 7). Consistent with the *NCED3* expression level, the ABA content was higher in the leaves of *CaREL1*-OX plants than in the leaves of wild-type plants.

## Discussion

Recently, several studies have reported that ABA-signalling components, which function as positive or negative regulators of abiotic stress responses, are ubiquitinated and degraded by E3 ubiquitin ligase and 26S proteasome, respectively^30, 31^. In the present study, we have demonstrated that *CaREL1* functions as a negative regulator of ABA sensitivity and drought tolerance. CaREL1 contains a RING-zinc finger domain in the C-terminal region; this domain is highly homologous with other C3H2C3 type RING E3 ubiquitin ligases found in diverse species (Fig. 1), including pepper (Supplementary Fig. S3). The presence of the RING finger domain in CaREL1 implies that this protein ubiquitinates target proteins. Ubiquitination is a post-translational modification process in eukaryotes, and it plays a key role in the recognition of and response to various hormone signals by controlling the protein stability^8, 32, 33^. To date, RING type E3 ligases have been associated with transferring ABA signals^24, 30, 34, 35^.

The expression of *CaREL1* was induced by ABA and drought stress (Fig. 2c), suggesting that this protein functions in the response to ABA-mediated drought stress. To determine the biological role of *CaREL1* in the ABA and drought stress responses, we generated *CaREL1*-silenced pepper plants and *CaREL1*-overexpressing Arabidopsis plants, because the transformation efficiency was very low in pepper plants. Detailed genetic analyses revealed that *CaREL1*-silenced pepper plants and *CaREL1*-OX Arabidopsis plants displayed drought-tolerant and drought-sensitive phenotypes, respectively. Moreover, these plants exhibited altered phenotypes in response to ABA. Generally, ABA sensitivity and drought tolerance in plants are determined by at least one cellular and molecular parameters, such as marker gene expression and stomatal aperture. Several studies have measured stomatal movement and established that high ABA sensitivity leads to increased drought tolerance^21, 25^. Consistent with the results of our phenotypic analysis in response to drought stress, *CaREL1*-silenced pepper plants and *CaREL1*-overexpressing Arabidopsis plants exposed to various ABA concentrations showed small and large stomatal pore sizes, respectively. Our data imply that the *in vivo* function of CaREL1 is related to the drought stress response, and that this is mediated by ABA-dependent signalling.

In the present study, we were unable to identify the substrate of CaREL1; however, the expression levels of ABA-dependent stress-related genes suggested that CaREL1 is involved in the ABA-dependent drought stress response. The results of a biochemical assay revealed that under drought stress conditions, ABA is more strongly accumulated in *CaREL1*-OX plants than in wild-type plants. Previous genetic studies have revealed that *aba1*, *aba2*, and *aba3* (ABA-deficient mutants) exhibited drought- and dehydration-sensitive phenotypes^36, 37^ characterized by reduced water potential and proline accumulation under water-deficit conditions^38^. Under these conditions, *NCED3* functions in ABA biosynthesis, which is crucial for the drought stress response in plants^39^. The up-regulation of ABA biosynthesis and *NCED3* expression in *CaREL1*-OX plants might be expected to amplify ABA-dependent defence signalling and therefore contribute to a drought-tolerant phenotype. In addition, *NCED3* expression positively modulates the transcription levels of stress-related genes, as well as the ABA level^40^. However, our genetic analyses revealed that *CaREL1*-OX plants exhibited a drought-sensitive phenotype characterized by ABA hyposensitivity (Figs 4 and 5) and decreased expression levels of ABA-dependent stress-related genes (Fig. 6). These findings cannot fully be explained by higher expression of *NCED3* and increased accumulation of ABA in *CaREL1*-OX plants. However, if CaREL1 is located upstream of ABA-dependent stress-related genes and inhibits defence response to drought stress mediated by these genes, the drought-sensitive phenotype of *CaREL1*-OX plants can be explained. Moreover, *CaREL1*-OX plants are unable to alleviate drought stress; hence, *CaREL1*-OX plants presumably recognize being in a stressful condition, leading to *NCED3* gene expression and ABA biosynthesis.

In conclusion, our results demonstrate that CaREL1 functions as a negative regulator of drought stress via altered sensitivity to ABA, as evidenced by changes in leaf temperature and stomatal apertures. We have demonstrated the involvement of CaREL1 in the defence responses to several abiotic stresses; however, the mechanism by which CaREL1 functions as a negative regulator of abiotic stress responses remains unclear. To elucidate the precise *in vivo* function of CaREL1 in response to abiotic stress, further studies to identify the substrates of this E3 ligase are required.

## Methods

### Plant materials

Seeds of pepper (*Capsicum annuum* L., cv. Hanbyul) and tobacco (*Nicotiana benthamiana*) were sown in a steam-sterilized compost soil mix (peat moss, perlite, and vermiculite, 5:3:2, v/v/v), sand, and loam soil (1:1:1, v/v/v). The pepper plants were raised in a growth room at 27 ± 1 °C under white fluorescent light (80 μmol photons·m^−2^·s^−1^; 16 h per day) as described previously^41^. The tobacco plants were maintained in a growth chamber at 25 ± 1 °C under a 16-h light/8-h dark cycle. *Arabidopsis thaliana* (ecotype Col-0) seeds were germinated on Murashige and Skoog (MS) salt (Duchefa Biochemie, Haarlem, Netherlands) supplemented with 1% sucrose and Microagar (Duchefa Biochemie); the seeded plates were incubated in a growth chamber at 24 °C under a 16-h light/8-h dark cycle. The Arabidopsis seedlings were maintained in a steam-sterilized compost soil mix (peat moss, perlite, and vermiculite, 9:1:1, v/v/v) under controlled environmental conditions as follows: 24 °C and 60% relative humidity under fluorescent light (130 μmol photons·m^−2^·s^−1^) with a 16-h light/8-h dark cycle. All seeds were vernalized at 4 °C for 2 days before being placed in the growth chamber.

### Sequence alignment and phylogenetic tree analysis

The encoded amino acid sequences for CaREL1 and its homologs were obtained using BLAST searches (http://www.ncbi.nlm.nih.gov/BLAST). The amino acid alignment was performed using ClustalW2 (http://www.ebi.ac.uk/Tools/msa/clustalw2), and the results were edited using Genedoc software (http://www.nrbsc.org/gfx/genedoc). The amino acid alignments were manually regulated to compare the cDNA clones of CaREL1 with those of other organisms. Based on the multiple sequence alignment data, a phylogenetic tree was drawn with MEGA software (version 5.2). To investigate sequence identity and similarity between two proteins, pairwise sequence alignment was performed using EMBOSS Needle webtool (http://www.ebi.ac.uk/Tools/psa/emboss_needle) with default parameters.

### Virus-induced gene silencing and overexpression of *CaREL1*

We used the tobacco rattle virus (TRV)-based virus-induced gene silencing (VIGS) system to generate *CaREL1* gene knockdown in pepper plants. We used the full-length *CaREL1* cDNA to generate *CaREL1-*overexpressing (OX) transgenic Arabidopsis plants, according to the protocol described previously^42^.

### ABA and drought treatments in pepper leaves

To examine the expression level of *CaREL1* in pepper leaves after ABA treatment, six-leaf-stage pepper plants were sprayed with 50 μM ABA. For the drought treatment, pepper plants were carefully removed from the soil to avoid injury. The plants were placed onto 3-mm filter paper (Whatman, Clifton, UK). Leaves were harvested 0–24 h after treatment and were subjected to RNA isolation and reverse transcription-polymerase chain reaction (RT-PCR) analysis.

To measure the rate of germination, root elongation, and seedling establishment, 36 seeds each of wild-type and *CaREL1-*OX transgenic Arabidopsis plants were stratified at 4 °C for 3 days and were then plated on 0.5× MS agar medium supplemented with various concentrations of ABA. The plates were incubated at 24 °C under white fluorescent light (130 μmol photons·m^−2^·s^−1^) with a 16-h light/8 h-dark cycle.

Three-week-old wild-type and *CaREL1-*OX seedlings were randomly planted and were then subjected to drought stress by withholding watering for 9 days and re-watering for 2 days. Survival rates were measured in each individual sample, and each experiment was performed three times with 30 plants. For pepper plants, drought stress was imposed on four-leaf-stage plants by withholding watering for 11 days. Plants were re-watered for 2 days to allow recovery, and the survival rate of the plants was then calculated. Survival rates were measured in each individual sample, and each experiment was performed three times with 20 plants. The drought resistance was determined in a quantitative manner by measuring the transpirational water loss. Fifty leaves were detached from four-leaf-stage pepper plants and 3-week-old Arabidopsis plants and placed in Petri dishes. The dishes were maintained in a growth chamber at 40% relative humidity, and the loss of fresh weight was determined at the indicated time points. All the experiments were performed at least in triplicate.

### Thermal imaging

For thermal imaging analysis, 4-week-old pepper plants having full expanded first and second leaves and 3-week-old Arabidopsis plants were treated with 50 μM ABA. Thermal images were obtained using an infrared camera (FLIR systems; T420) and the leaf temperature was measured with FLIR Tools+ ver 5.2 software.

### Stomatal aperture

To measure the stomatal aperture, epidermal peels were stripped from rosette leaves of 3-week-old plants and floated in a stomatal opening solution (SOS: 50 mM KCl and 10 mM MES-KOH, pH  6.15, 10 µM CaCl_2_) in the light. After incubation for 3 h, the buffer was replaced with fresh SOS containing 10 μM or 20 μM ABA. After an additional 2 h of incubation, stomatal apertures were measured in each individual sample, and each experiment was performed three times with 20 leaves.

### RNA isolation and semi-quantitative and quantitative reverse transcription-polymerase chain reaction

Total RNA was isolated from the Arabidopsis leaf tissues, which were dehydrated or infected with the bacterial pathogen using an RNeasy Mini kit (Qiagen, Valencia, CA, USA). To remove genomic DNA, all RNA samples were digested with RNA-free DNase. After quantification using a spectrophotometer, 1 μg of total RNA was used to synthesize cDNA using a Transcript First Strand cDNA Synthesis kit (Roche, Indianapolis, IN, USA) according to the manufacturer’s instructions. Concomitantly, cDNAs were synthesized without reverse transcriptase and were subjected to semi-quantitative RT-PCR to rule out the possibility of contamination by genomic DNA in the cDNA samples. For quantitative reverse transcription-polymerase chain reaction (qRT-PCR) analysis, the synthesized cDNA was amplified in a CFX96 Touch^TM^ Real-Time PCR detection system (Bio-Rad) with iQ^TM^ SYBR Green Supermix and specific primers (Supplementary Table S1). Every reaction was performed in triplicate. The PCR was programmed as follows: 95 °C for 5 min; 45 cycles each at 95 °C for 20 s and 60 °C for 20 s; and 72 °C for 20 s. The relative expression of each gene was calculated using the ∆∆Ct method, as described previously^43^. The Arabidopsis *actin8* gene (*AtACT8*) was used for normalization.

### *In vitro* ubiquitination

The procedure for the expression and purification of the maltose-binding protein (MBP)–CaREL1 recombinant protein is described in Park *et al*.^42^. For the *in vitro* ubiquitination assay, the purified MBP–CaREL1 (500 ng) was mixed with ubiquitination reaction buffer [50 mM Tris-HCl, pH 7.5, 10 mM MgCl_2_, 0.05 mM ZnCl_2_, 1 mM Mg-ATP, 0.2 mM DTT, 10 mM phosphocreatine, and 0.1 unit of creatine kinase (Sigma-Aldrich)] containing 250 ng of recombinant human UBE1 (Boston Biochemicals, Cambridge, MA, USA), 250 ng of recombinant human H5b (Enzo Life Sciences, Farmingdale, NY), and 10 μg of bovine ubiquitin (Sigma-Aldrich). After incubation at 30 °C for 3 h, the reacted proteins were separated using SDS-PAGE and analysed using immunoblotting with anti-ubiquitin antibody (Santa Cruz Biotechnology, Santa Cruz, CA) and anti-MBP antibody (New England Biolabs, Ipswich, MA).

### Measurement of ABA content

For determination of ABA content, leaves were harvested from pepper and Arabidopsis plants treated with dehydration for 2 and 4 h and immediately frozen in liquid nitrogen. Approximately 50 mg of ground tissue were extracted overnight in 1 ml of ABA extraction buffer (methanol, containing 100 mg butylated hydroxyl toluene, 0.5 g citric acid monohydrate) at 4 °C on a rotary shaker. After centrifuged at 1500 g, the supernatant was transferred to new tube and dried using a speed vac. ABA content of each sample was quantified by using the Phytodetek-ABA kit (Agdia Inc., Elkhart, IN, USA) according to manufacturer’s instruction. ABA contents were expressed as pmol mg^−1^ fresh weight of the tissue.

### Statistical analyses

To determine significant differences between different plant lines in response to treatments, statistical analyses were performed using one way analysis of variance (ANOVA) or student’s t-test. A P value of <0.05 was considered significant.

## Electronic supplementary material

Supplementary data
