# Supplementary material for: Pepper CaREL1, a ubiquitin E3 ligase, regulates drought tolerance via the ABA-signalling pathway
Source: Sci Rep. 2017 Mar 28;7:477. doi: 10.1038/s41598-017-00490-4 (PMC5428412; doi:10.1038/s41598-017-00490-4)
Supplement: Supplementary file 1 — Supplementary data [file 41598_2017_490_MOESM1_ESM.pdf]

Supplementary data

Pepper CaREL1, a ubiquitin E3 ligase, regulates drought tolerance via the ABA-signalling pathway

Chae Woo Lim, Chanmi Park, Jung-Hyun Kim, Hyunhee Joo, Eunji Hong, Sung Chul Lee

Supplementary Table S1. Sequences of primers used in this study

Primer name	Primer sequence (5'-3')
For cloning	
CaREL1	Forward: ATGGGTAATTGTTGCTGCTG
	Reverse: TCCATCGATTGCAGGGTTA
CaREL1$^{C205S/C208S}$	Forward: CGTTTCCTGATCACTGACGGGGCTGGTTTCACTTCTCT
	Reverse: AGAGAAGTGAAACCAGCCCCGTCAGTGATCAGGAAACG
For RT-PCR	
CaREL1	Forward: CTCGAGCATCTCATCATGTCACAGC
	Reverse: TCTAGAAGTTTCACTGTTTGTTTCTTGA
CaACT1	Forward: GACGTGACCTAACTGATAACCTGAT
	Reverse: CTCTCAGCACCAATGGTAATAACTT
Actin8	Forward: CAACTATGTTCTCAGGTATTGCAGA
	Reverse: GTCATGGAAACGATGTCTCTTTAGT
NCED3	Forward: ACATGGAAATCGGAGTTACAGATAG
	Reverse: AGAAACAACAAACAAGAAACAGAGC
DREB2A	Forward: CTACAAAGCCTCAACTACGGAATAC
	Reverse: AAACTCGGATAGAGAATCAACAGTC
RAB18	Forward: GGAAGAAGGGAATAACACAAAAGAT
	Reverse: GCGTTACAAACCCTCATTATTTTTA
RD20	Forward: TGGTTTCCTATCTAAAGAAGCTGTG
	Reverse: ATACAAATCCCCAAACTGAATAACA
RD29B	Forward: GTTGAAGAGTCTCCACAATCACTTG
	Reverse: ATACAAATCCCCAAACTGAATAACA
RD29A	Forward: CACAATCACTTGGCTCCACTGTTG
	Reverse: ACCTAGTAGCTGGTATGGAGGAACT
KIN2	Forward: TGTTAACTTCGTGAAGGACAAGAC
	Reverse: ACAACAACAAGTACGATGAGTACGA
ABI1	Forward: GTTTGGGATGTAATGACGGATG
	Reverse: TGAACTGAGGCAGAGAGGGTCC
ERD1	Forward: CCACCGACTCCTCTCTGCTT
	Reverse: AAGGGAGATTCCGAGATATGAAGA

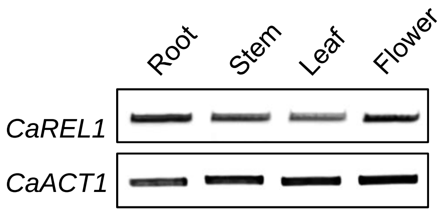

Supplementary Figure S1. RT-PCR analysis of *CaREL1* expression in various tissues of pepper plants.

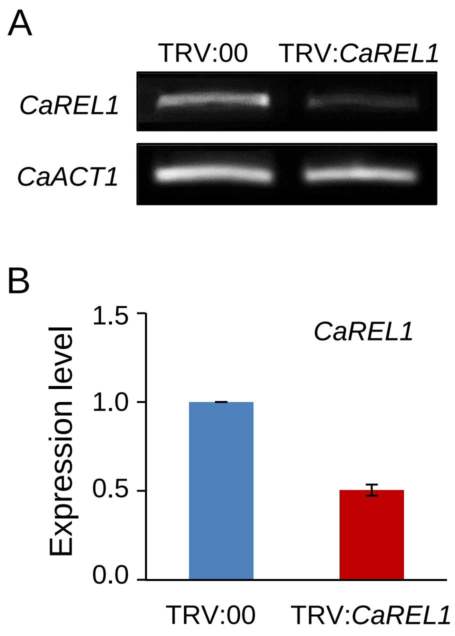

Supplementary Figure S2. RT-PCR (A) and qRT-PCR (B) analysis of *CaREL1* expression in the leaves of pepper plants transfected with the empty vector control (TRV:00) or *CaREL1*-silenced constructs (TRV:*CaREL1*). The expression levels were measured 0 h after detachment. The *Actin1* gene was used as an internal control..

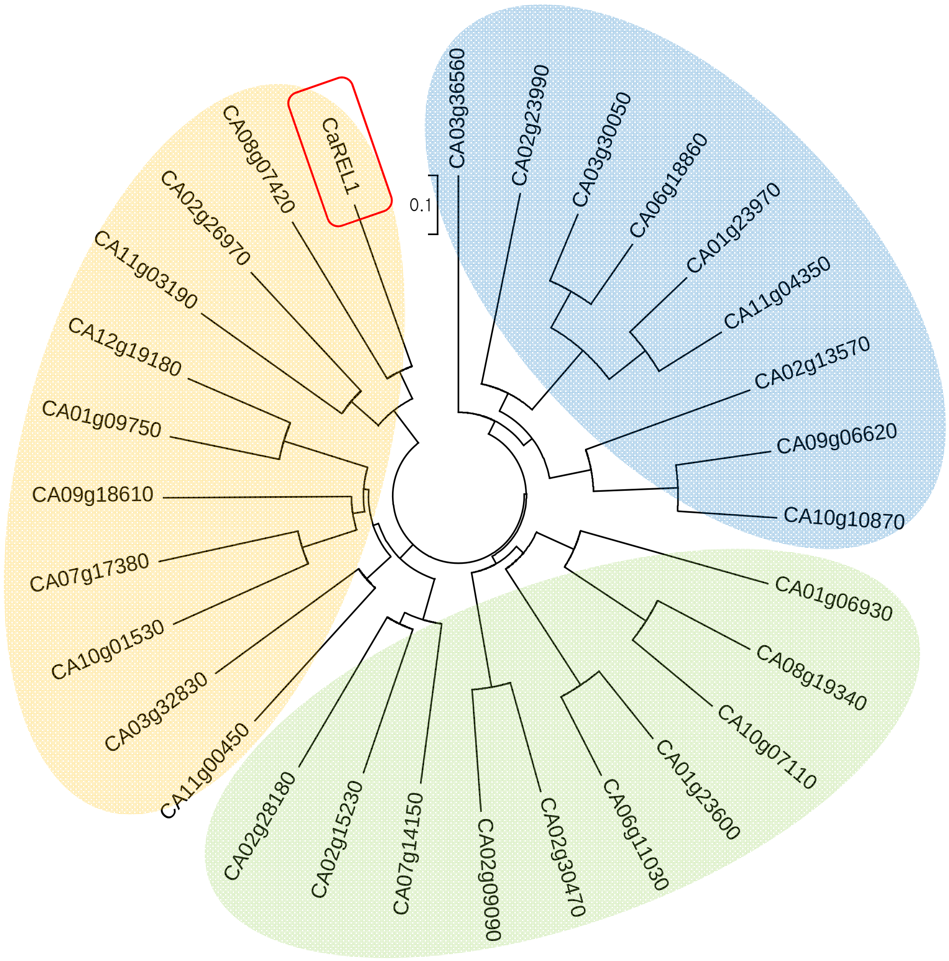

Supplementary Figure S3. Multiple alignment of amino acids in the CaREL1 protein and its homologous pepper proteins was performed using ClustalW2.
